# Providing Mental Health First Aid Training to Hatzola

**DOI:** 10.1192/bjo.2022.112

**Published:** 2022-06-20

**Authors:** Ailbhe Brennan, Jack Hubbett, Rosalyn Buckland, Hugh Grant-Peterkin

**Affiliations:** 1 East London NHS Foundation Trust, London, United Kingdom; 2 Royal Free London NHS Foundation Trust, London, United Kingdom

## Abstract

**Aims:**

Hackney is home to the largest Charedi Orthodox Jewish community in Europe. According to the Census 2011, 7% of the population of Hackney are Charedi. Hatzola is a non-profit, volunteer organisation established in 1979 to provide pre-hospital emergency medical response and transportation to acute hospitals at no cost, to those living in and around the North London Charedi community. Given the large Charedi population served by Homerton University Hospital it is a common occurrence for psychiatry liaison staff to work side by side with Hatzola in delivering care to those in mental health crisis. Our aim was to create and nurture a professional relationship between Homerton University Hospital Psychiatry Liaison Service and Hatzola ambulance. We wanted to gain an understanding of the perception of mental illness within the Charedi community, and identify issues faced by members of Hatzola when working with those with mental illness. We wanted to identify the learning needs of Hatzola around psychiatric illness as well as increasing confidence within team members when called to manage mental health crises.

**Methods:**

We scheduled an initial meeting with Hatzola to gain an understanding of their service. We used questionnaires to ascertain their level of knowledge on managing mental health patients. We set out to provide teaching sessions to address Hatzola's learning needs.

We designed interactive teaching sessions based on providing mental health first aid, discussing case studies, considering the legal framework around emergency mental health. We ensured coverage of working with both adults and children with mental health difficulties. We delivered these teaching sessions in person over four consecutive weekly meetings, with the sessions being recorded to serve as an educational resource.

**Results:**

We gathered qualitative evidence reflecting the impact of our intervention. We were able to compare levels of confidence among Hatzola members before and after our teaching programme.

**Conclusion:**

Our training programme was well received by Hatzola, and it was an excellent opportunity to develop links with members of the community.

We have learned that mental health is a taboo subject for members of the Charedi community, and have identified a need for more support to Hatzola in coping with the emotional toll working with mental health patients can take. There may be scope for providing further training on developing reflective practice and more emotional support for Hatzola members in future.

